# Catatonia in Obsessive-Compulsive Disorder: A case study and literature review

**DOI:** 10.1192/j.eurpsy.2022.1651

**Published:** 2022-09-01

**Authors:** A. Suddath, A. Franks, H. Winston

**Affiliations:** University of Colorado, Psychiatry, Aurora, United States of America

**Keywords:** Catatonia, OCD, autoimmune

## Abstract

**Introduction:**

There are extremely few reported cases of OCD causing catatonia and some of those cases are possibly associated with the somewhat contentious diagnosis of Pediatric Autoimmune Neuropsychiatric Disorder Associated with Streptococcus. As there is a symptom overlap between OCD and catatonia some cases of catatonia are possibly being missed, warranting discussion regarding differential diagnosis, symptomatology, and treatment of catatonia and OCD.

**Objectives:**

We describe a case of a 18-year-old patient who developed severe catatonia secondary to OCD, possibly related to PANDAS/PANS. We discuss the complex work-up, differential diagnosis, and treatment of this patient.

**Methods:**

Discussion of a single case and a review of catatonia literature as it relates to OCD and autoimmune disorders.

**Results:**

Our patient was an 18-year-old Ukrainian male who presented with sub-acute onset of decreased movement, decreased oral intake, and inability to speak. He was diagnosed with catatonia of an unclear etiology and treated with high-dose lorazepam at an outside hospital then transferred to our care. Presenting symptoms were then clarified and found to be consistent with OCD, upon which OCD treatment was initiated. The patient’s sub-acute and severe onset of OCD raised the question of a PANDAS/PANS diagnosis, which was further investigated. Ultimately, his symptoms improved with ongoing lorazepam and he was transferred to another hospital for ECT treatment.

**Conclusions:**

OCD has been observed to cause catatonia in extremely rare cases. Diagnosing catatonia associated with OCD is challenging and important as catatonia is associated with significant morbidity and mortality if left untreated. Our patient improved with concurrent treatment of catatonia and OCD.

**Disclosure:**

No significant relationships.

